# German Language Acquisition of Refugee Children—The Role of Preschools and Language Instruction

**DOI:** 10.3389/fsoc.2022.840696

**Published:** 2022-05-09

**Authors:** Julian Seuring, Gisela Will

**Affiliations:** Leibniz Institute for Educational Trajectories (LIfBi), Bamberg, Germany

**Keywords:** language acquisition, refugees, preschool, language instruction, Germany

## Abstract

Mastering the language of the destination country is key to immigrant and refugee children's educational success. Refugee children typically face the challenge of starting or continuing their educational carrier in a completely new context and in a completely new language. In this study, we examine the role of preschool attendance and formal language instruction in supporting young refugee children to acquire destination language competencies. We pursue three research objectives: *First*, we aim at identifying relevant conditions associated with German language acquisition in general. *Second*, we examine the (relative) importance of institutional learning support in preschool and language instruction. *Third*, we investigate whether the benefits of attending preschool are more pronounced for refugee children who have only limited exposure to the destination language outside of the institutional context, as compared to children who have more exposure to the language outside of preschool. Using data from the ReGES study, we analyze the early processes of destination language acquisition among a large population of refugee children of preschool age in Germany. Our findings indicate that conditions associated with motivation, exposure and efficiency of learning that were found in prior research to determine destination language competencies of children from other immigrant groups apply to refugee children in a similar manner. Additional conditions associated with the specific circumstances that refugees often experience, including possible consequences of insecure residence status, risk of post-traumatic stress disorders, and living in collective accommodation, do not significantly contribute to this outcome in our analysis. Furthermore, we find that there is a positive relationship between children's German language competency levels and both preschool attendance and formal language instruction. The findings indicate that the benefits of attending preschool are largely related to additional language instruction that refugee children receive within this context. Moreover, these benefits are particularly pronounced among refugee children who have only limited exposure to German at home and in their everyday lives. Overall, our findings emphasize the importance of preschool attendance and formal language instruction for refugee children's destination language acquisition.

## Introduction

Learning the language of the destination country is key to immigrants' incorporation into the host society. For the children of immigrants, mastering the destination language—which is also the language of instruction in school in most countries—is a crucial factor in building a successful educational career (Schnepf, 2007; Alba et al., 2011; Azzolini et al., 2012). Language barriers can prevent immigrant children from reaching their full educational potential, and can shape ethnic inequalities in school (Heath et al., 2008; Diehl et al., 2016). Recent studies indicate that this key finding from prior research on the children of immigrants also applies more generally to the educational integration of refugee children (e.g., Maué et al., 2021; Schipolowski et al., 2021). Against this backdrop, the conditions that foster or hinder the destination language acquisition of refugee children gain additional relevance.

Refugees migrate under specific circumstances which set them apart from those immigrants who migrate for primarily economic or family reasons. Many refugees experienced traumatic events in their home countries, or during their journeys, as well as interruptions of their educational biographies, and uncertain prospects when it comes to their right to stay in their destination countries. Moreover, they have been forced to leave their homes, often with little preparation for their flight or their future lives in their destination countries. A major challenge for refugees' integration is, for example, that they usually enter countries without prior knowledge of the languages spoken there. Accordingly, in the research, it is currently being debated whether the factors that facilitate language acquisition, that have been identified for other immigrant populations also hold for the integration processes of refugees, as well as whether additional factors that are associated with the specific experience of refugees become (more) important (FitzGerald and Arar, 2018; Kogan and Kalter, 2020).

Concerning destination language acquisition, findings from prior studies consistently indicate that the main factors underlying the development of destination language competencies do not differ between *adult* refugees and other immigrant groups (e.g., Fennelly and Palasz, 2003; Hou and Beiser, 2006; Van Tubergen, 2010; Kosyakova et al., 2021; Kristen and Seuring, 2021). However, structured learning opportunities such as taking language instruction appear to be particularly beneficial for refugees (Kosyakova et al., 2021; Kristen and Seuring, 2021). Prior findings also indicate that formal language instruction is most efficient in the early stages of the learning process (e.g., Hoehne and Michalowski, 2016; Bernhard and Bernhard, 2021). In light of these findings, it can be assumed that young refugee *children*, who typically learn the target language from scratch upon arrival in the destination country, benefit greatly from institutional language instruction—instruction which takes place mainly in the context of preschool education for young children (Will et al., 2018).

In this study, we investigate the role of preschool attendance and language instruction in the language acquisition of young refugees, namely children of preschool age in Germany. Specifically, we pursue three research objectives. *First*, we aim at identifying the factors that underlie the German language acquisition of refugee children in general. *Second*, we examine the (relative) importance of institutional learning support in preschool and language instruction. *Third*, we analyze whether the benefits of structured learning opportunities in preschool are particularly pronounced among children who have only limited exposure to the target language outside of the institutional context, for instance, within their families.

Starting from a general theoretical model of destination language acquisition (Chiswick and Miller, 1995, 2001; Esser, 2006a,b), we investigate various conditions that are associated with the overarching factors behind language acquisition, which are motivation, exposure, and efficiency. Building on the notion that integration processes are characterized by regularities that affect refugees and other immigrant groups in a similar way (Kogan and Kalter, 2020), we hypothesize that the main conditions that have been found to shape language acquisition among the children of immigrants also hold for refugee children. Such conditions include, among others children's general cognitive abilities, and their degree of contact with the destination language in their everyday lives (Becker, 2007). For refugees, however, additional conditions might become relevant, which are associated with specific characteristics that set them apart from other immigrants (Kristen and Seuring, 2021). For example, most refugees have spent time living in welcome centers and group accommodation upon arrival in the destination country and would have had only limited exposure to the destination language within these contexts (e.g., Van Tubergen, 2010). Another factor that is frequently discussed is refugees' experiences of traumatic events and the resulting mental health issues that could affect language acquisition (e.g., Hunkler and Khourshed, 2020).

Besides the effects of individual conditions, access to institutional programs and other opportunities for language support can have an impact on destination language acquisition. Prior studies indicate that attending preschool supports immigrant children in learning the destination language, particularly those children who have only limited linguistic exposure outside of the institutional context (e.g., Magnuson et al., 2006; Gormley, 2008; Becker et al., 2013; Klein and Becker, 2017). This tendency is likely to also apply to refugee children, who often stay in collective accommodation with their parents, and have little exposure to the German language outside of institutional programs. However, despite the assumed advantages of language support, refugee children in Germany attend preschool less often than their majority peers (Homuth et al., 2021) and rarely take language instruction (Will et al., 2018). Identifying the importance of institutional language support for refugee children's destination language acquisition could thus also reveal possible target points for interventions.

Prior research has mainly focused on children of immigrants who moved for economic or family reasons, whereas the situation of refugee children has rarely been addressed. Apart from the special circumstances associated with their flight, refugee children also offer research studies a different focus. Whereas the vast majority of prior studies have focused on the so-called second and third generations, i.e., children who were born in Germany and whose parents or even grandparents were born abroad, refugee children actually constitute a generation that has migrated itself. Using data from the ReGES survey (“Refugees in the German Educational System”; Will et al., 2021), we extend the scope of prior research and examine the early processes of destination language acquisition among a large population of first generation refugee children. Our study contributes to existing literature in several regards: we focus on the situation of children in a very early stage of their education, i.e., before they enter primary school, and can identify conditions that could cause ethnic inequalities right from the start of children's educational career. In our analysis, we can rely on the results of standardized language tests (vocabulary and grammar), while most other studies on refugees examined respondents' self-reports of their language proficiency, which have been shown to be less reliable, and in some instances, biased (Edele et al., 2015). Moreover, we investigate whether language learning in preschools mainly occurs through formal language instruction programs offered within the institutions, or whether other factors are also important, such as general contact with the target language in this setting. The benefits of learning support in preschools could differ according to children's levels of target language exposure in their everyday lives outside of the institutional settings. Identifying such tendencies might reveal additional target points for institutional support, especially for those in need.

## Language Acquisition in the Destination Context

### A General Model of Destination Language Acquisition

According to a human capital approach of destination language acquisition (Chiswick and Miller, 1995, 2001), the level of language competencies that immigrants achieve is the outcome of a series of investment decisions to engage in activities that foster language learning (Espenshade and Fu, 1997; Esser, 2006a,b; Kristen, 2019). Such investments include deliberate activities to improve language competencies (e.g., taking language instruction), as well as activities that are not specifically aimed at increasing language competencies but do so as a side-effect. These include, for example, everyday interactions with majority members (Kristen et al., 2016). When it comes to young children, language learning is likely to proceed unconsciously, and is not driven by active investment decisions on the part of children. In this case, investments in language competencies are often made by the parents or educators (Chiswick and Miller, 1995), but the underlying mechanisms of learning follow the same principles (Esser, 2006a,b).

In the general model, immigrants' acquisition of the destination language is related to three overarching factors that determine investments in language learning activities: the *incentives* associated with improved language competencies, the *exposure* to that language—in terms of both quantity and quality of language input—and the learning *efficiency*, which reflects an individual's cognitive capacity for processing the available input (Chiswick and Miller, 1995, 2001).

Given its general nature, the model has been applied in past research across disciplines, immigrant groups, immigrant generations, age groups, and competence domains. The model provides an analytical framework to systematically relate individual and contextual conditions to language acquisition processes. Prior research has identified various conditions associated with the three overarching factors of destination language acquisition (for an overview, see Kristen, 2019). In the following, we briefly discuss the conditions that are most relevant for studying destination language acquisition among immigrant and refugee children.

Whereas the initial model stresses the importance of economic *incentives* associated with the development of language competencies (Chiswick and Miller, 1995, 2001), adaptions of the model to other disciplines also address the non-economic benefits of learning a new language (e.g., Espenshade and Fu, 1997; Esser, 2006a,b). Although economic incentives, such as future wages of children, could (indirectly) play a role in parents' investment in their children, other aspects that more directly determine children's everyday lives are a major driver for language learning; for instance, improving destination language competencies can promote children's educational performance (e.g., Schnepf, 2007; Alba et al., 2011; Azzolini et al., 2012) and may help them to establish contact with majority peers (e.g., Martinovic et al., 2009; Schacht et al., 2014). In line with this reasoning, we apply a broader concept of incentives, encompassing all conditions that motivate individuals to engage in learning activities. In the following, we refer to the term *motivation* (Esser, 2006a,b) to indicate both economic and non-economic incentives.

Immigrants' intention to stay in the destination country can shape their *motivation* to learn the destination language. The returns from language investments increase with the duration immigrants stay in the destination country (Chiswick and Miller, 2001). Moreover, some returns can only be achieved in the long term, such as educational qualifications associated with improved language competencies. Accordingly, immigrants and their children who intend to stay in the destination country (for a longer period) are more inclined to pursue high levels of language competencies compared to families with re-migration intentions. However, conditions associated with immigrants' motivation have barely been touched upon in prior studies (Kristen, 2019). The few studies that have examined the relationship between the intention to stay and destination language competencies yielded inconclusive results (e.g., Kristen et al., 2016; Bernhard and Bernhard, 2021; Kosyakova et al., 2021; Kristen and Seuring, 2021).

*Efficiency* refers to an individuals' ability to translate the available input into improved language competencies. More efficient learners are expected to improve their language competencies faster and become more proficient in the long-run (Van Tubergen and Mentjox, 2014; Kristen, 2019). Learning efficiency is largely determined by individuals' general cognitive abilities, which have also been found to significantly predict immigrant children's language competency levels in prior research (e.g., Becker, 2007; Seuring et al., 2020).

*Exposure* to the destination language is another major factor for learning. Being exposed to contexts in which the destination language is frequently used can help immigrants to improve their language competencies. Opportunities to come into contact with the destination language can occur both in formal settings, such as at school or in language courses, as well as in everyday situations, for example through everyday interactions with locals, or the consumption of media (Stevens, 1992; Chiswick and Miller, 1995; Van Tubergen and Mentjox, 2014; Kristen, 2019). The longer they stay in the destination country, the more exposure to the destination language immigrants accumulate, thereby gaining proficiency (Van Tubergen, 2010). In line with this, prior findings consistently indicate that destination language competencies improve with a longer duration of stay (e.g., Chiswick and Miller, 1995; Espenshade and Fu, 1997; Stevens, 1999; Van Tubergen and Kalmijn, 2005; Braun, 2010; Bernhard and Bernhard, 2021; Kosyakova et al., 2021; Kristen and Seuring, 2021). Furthermore, previous studies clearly indicate that destination language use within the family, with friends and other persons, for example, at work or school fosters its acquisition (e.g., Espenshade and Fu, 1997; Stevens, 1999; Braun, 2010; Kristen et al., 2016; Seuring et al., 2020; Bernhard and Bernhard, 2021; Kosyakova et al., 2021; Kristen and Seuring, 2021). For young children, language input and support within the family is crucial for their destination language acquisition. Parents' own competencies in the destination language and regular use of that language within the family have been identified as favorable conditions for children's language acquisition (Becker, 2007, 2011). In addition to informal language input, participation in language courses was found to promote the language learning of adult immigrants (e.g., Van Tubergen, 2010; Kosyakova et al., 2021; Kristen and Seuring, 2021), especially when instruction takes place in an early stage of the learning process (e.g., Hoehne and Michalowski, 2016; Bernhard and Bernhard, 2021). It can be assumed that these tendencies also apply to immigrant children. In addition, attending preschool was found to significantly support immigrant children in learning the destination language, and particularly those children who receive only limited language input at home (e.g., Magnuson et al., 2006; Gormley, 2008; Becker et al., 2013; Klein and Becker, 2017).

### Destination Language Acquisition Among Refugee Children in Germany

The circumstances of refugee migration are markedly different from those of voluntary immigrants, who are motivated by economic or family reasons. Refugees have been forced to leave their home country and have experienced extraordinary situations prior to, during, and after migration, making refugees an immigrant population with special features. However, despite refugees' specific experiences, we subscribe to the view that integration processes undergo regularities that apply to all immigrants (including refugees) in the same way (Kogan and Kalter, 2020). Accordingly, we hypothesize that the previously discussed conditions associated with motivation, efficiency and exposure also determine the success of destination language acquisition among refugee children. Specifically, we expect that the intention to stay in the destination, general cognitive abilities, the duration of stay, language input at home and in everyday life, language instruction, and attendance of preschool all positively relate to refugee children's destination language competencies.

While the basic processes underlying destination language acquisition should not differ, some additional conditions might become relevant for refugees which are associated with specific characteristics that set them apart from other immigrants (Kristen and Seuring, 2021). Such peculiarities do not represent distinct mechanisms, but relate to individual and contextual conditions that empirically seldom apply to other immigrant groups (Kogan and Kalter, 2020). Thus, refugee-specific conditions that affect destination language acquisition can also be linked to motivation, efficiency and exposure.

Refugees' legal status constitutes a prominent example for a specific condition shaping their motivation for learning the destination language. The formal process of applying for a residence permit in Germany can be lengthy, and until a final decision is made, refugees cannot be sure about their prospects of remaining in the country (Kosyakova and Brenzel, 2020). Such uncertainties can discourage refugees from investing in integration activities (Hvidtfeldt et al., 2018; Kosyakova and Brenzel, 2020), such as learning the destination language (Van Tubergen, 2010; Kosyakova et al., 2021; Kristen and Seuring, 2021). Parents without secure prospects of staying may also be more reluctant to support the destination language acquisition of their children and, for example, may see no need to send them to preschool.

Another conditions that is frequently discussed is refugees' experiences of traumatic events and related mental health issues that could affect language learning (e.g., Hunkler and Khourshed, 2020). Mental health problems are expected to curb individuals' efficiency in learning a new language (Chiswick and Miller, 2001; Van Tubergen and Kalmijn, 2005). Previous studies, however, have not found support for a negative association between traumatic experiences or poor mental health and destination language competencies among adult refugees (e.g., Van Tubergen, 2010; Hunkler and Khourshed, 2020; Bernhard and Bernhard, 2021; Kosyakova et al., 2021; Kristen and Seuring, 2021). However, whether individuals have experienced traumatic events at younger age or later in life, could make a difference, assuming that children and adolescents process traumatic experiences differently than adults. Some studies, for instance, have found a negative association between post-traumatic stress disorder symptoms and refugees' educational performance in school (e.g., Will and Homuth, 2020).

In Germany, most refugees stay in collective accommodation organized by the authorities for several months after their arrival (Brücker et al., 2020). Families living in collective accommodation together with many other refugees may only have limited exposure to the destination language (Van Tubergen, 2010). In such a shielded living environment, refugees do not have many opportunities to have contact with majority members and learn the destination language through everyday interactions (Kosyakova et al., 2021). According to this reasoning, living in collective accommodation for refugees should hamper both the language acquisition of parents and their children. In some instances, however, collective accommodation could also provide a beneficial environment for language learning. For example, if refugee families from many different language backgrounds share the same accommodation, children are more inclined to use the destination language to communicate with each other. In addition, children in group accommodation may have a better access to language instruction programs, which are sometimes specifically promoted in such facilities.

### The Institutional Context of Preschools and Language Instruction

In order to support refugees in their early integration processes, the German government considerably increased its expenditures on institutional language instruction programs specifically designed for adult refugees (Brücker et al., 2019; Kosyakova and Brenzel, 2020). Although all immigrants should benefit from taking language instruction, prior findings indicate that institutional language instruction is particularly effective for refugees (e.g., Kosyakova et al., 2021; Kristen and Seuring, 2021). In light of findings indicating that formal language support is more efficient in an early stage of the learning process (e.g., Hoehne and Michalowski, 2016; Bernhard and Bernhard, 2021), young refugee children should benefit greatly from language instruction.

For young children, language instruction mainly takes place in the context of preschool education (Will et al., 2018). In Germany, according to § 24 SGB VIII, from the age of one children have a legal right to institutional childcare. This law generally also applies to immigrant and refugee children living in Germany, regardless of their residence status and their prospects of staying (Meysen et al., 2016). Children up to the age of three typically attend daycare or other types of childcare, whereas children aged 3–6 attend preschool. Despite this legal framework, some families still have difficulties finding childcare because there are not enough options, especially with regard to daycare for children under the age of three (BMFSFJ, 2018). Moreover, the shortage of childcare options varies greatly from region to region (BMFSFJ, 2018). Finding childcare could be particularly difficult for newly immigrated families, since these services often need to be expanded to meet the growing demand associated with an increased immigration. A shortage of available childcare options, could be one of the reasons why refugee children in Germany attend preschool less often than their majority peers (e.g., Homuth et al., 2021). Expanding childcare options on a larger scale could be an effective tool to increase overall participation in daycare, and especially the attendance of immigrant and refugee children (see e.g., Roth and Klein, 2018).

Apart from the continuous expansion of childcare options, there are also efforts in Germany to improve the quality of childcare, for example, by promoting the language development of children. As part of the “Act on good early childhood education and care” (KiQuTG) program, children should be supported in developing basic language and literacy skills during everyday care in preschool (BMFSFJ, 2020). In addition, preschool often provides language instruction specifically aimed at supporting children with language difficulties, for example, immigrant children (Becker et al., 2016). While support for basic language development is typically implemented in activities of regular care, language instruction for those in need takes place as an extracurricular activity (BMFSFJ, 2020). However, whether preschool institutions offer specific language instruction for immigrant and refugee children, and how such programs are implemented depends strongly on the institutional context, and varies between federal states and regions. For example, preschools with a high share of immigrant children more often offer language instruction programs, compared to institutions with fewer immigrants (BMFSFJ, 2020).

Previous studies consistently indicate positive effects of general preschool attendance on children's language acquisition (e.g., Sammons et al., 2004; Magnuson et al., 2006; Loeb et al., 2007; Gormley, 2008; Burger, 2012; Becker et al., 2013; Klein and Becker, 2017), whereas the role of language instruction provided in these contexts has rarely been addressed empirically (for exception see e.g., Becker et al., 2016). It is, thus far, an open question whether language learning in preschool occurs largely through formal language instruction offered within the institutions, or through the increased contact with the destination language that children experience in general care, for example, during interactions with peers and educators. It can be expected that both aspects significantly promote the destination language acquisition of children attending preschool.

For recently settled refugee children, who often speak hardly any German when they enter preschool, language instruction might be an important prerequisite for communicating with other children and educators. Thus, refugee children who receive additional language instruction should profit more from attending preschool than those who are only in general care. Moreover, prior research has shown that formal language instruction among adult refugees on the one hand, and preschool attendance among immigrant children on the other hand, both particularly favor the learning process of individuals who have only limited exposure to the destination language outside the institutional context (e.g., Magnuson et al., 2006; Gormley, 2008; Becker et al., 2013; Klein and Becker, 2017; Kristen and Seuring, 2021). It is probable that this consistent pattern also applies to the situation of refugee children in preschool. Accordingly, refugee children with little exposure to the destination language in their everyday lives and at home can particularly benefit from attending preschool.

## Data and Methods

### Description of ReGES-Data and Analysis Sample

To analyze our research questions, we use data from the study ReGES—Refugees in the German Educational System (see Will et al., 2021)^1^. In ReGES, two cohorts of young refugees were sampled at different stages of their educational career: children aged four or older who had not yet started school (Refugee Cohort 1), and adolescents who were attending lower secondary education in the German school system (Refugee Cohort 2). For our analysis, we exclusively focus on data of the first survey wave of Refugee Cohort 1, which was conducted in spring 2018. The ReGES study was conducted in five federal states (Bavaria, Hamburg, North Rhine-Westphalia, Rhineland-Palatinate and Saxony; for details on sampling see Steinhauer et al., 2019). Within these federal states, a sample of refugee children was drawn from registration data across selected municipalities. Sampling was restricted to children who were citizens of those countries from which the highest numbers of refugees had come to Germany in the mid-2010s, and who had good prospects of staying. Before participating in the survey, a screening interview was conducted to determine whether the children actually belonged to the target population. They had to meet five criteria: (1) their asylum application was in progress or at least planned, (2) they immigrated to Germany on January 1, 2014 or later, (3) they lived together with a parent or legal guardian, (4) they had been in Germany for at least 3 months, and (5) they were minimum 4 years old, but had not started school. The interviews were conducted face-to-face with one of the children's parents or legal guardians. Only refugees who spoke one of the eight interview languages (apart from German, these were Arabic, English, Farsi, French, Kurmanji, Pashto, and Tigrinya) were able to participate in the survey.

In addition to this selection of participants for the first survey wave, the subsequent panel waves were administered in only four languages (Arabic, German, English, and Kurmanji) for practical reasons. From the *N* = 2,405 children for which information was collected in the first wave, *N* = 2,251 children were followed in the panel. Among participants of the panel population, a more extensive set of information was assessed during data collection for the first survey wave. For instance, an additional survey of the educational staff in preschool was conducted (Heinritz and Will, 2021), and the target children's basic cognitive competencies and German language competencies (vocabulary and grammar) were assessed using technology-based tests. Testing required some basic command of one of the panel languages in order to understand the instructions, which was the case for the majority of children (*N* = 2,199). In our analysis, we include only children who obtained a valid score in the German vocabulary test. Children who did not participate in testing (*N* = 191), who canceled the test, for example because of technical issues (*N* = 406), and those who did not pass the screening test, or scored too low to obtain a valid assessment (*N* = 261) had to be excluded. The final analysis sample consists of *N* = 1,341 children and their parents.

### Variables and Measures Used for Analysis

The children's level of *German language competencies* were assessed using the “Peabody Picture Vocabulary Test” (PPVT-4; Dunn and Dunn, 2007) in its German version (Lenhard et al., 2015; for details on its use in ReGES see Obry et al., 2021). In the test, single words were played from a recording, which the children then had to correctly assign to one of four pictures. The test consisted of 19 sets with 12 items each and a total of 228 items. The test was administered by proceeding from easier to harder sets thereby steadily increasing the difficulty. If children solved fewer than five items of a set correctly, the test was stopped at this level, because it was assumed that subsequent sets would be too challenging. The test scores are used as the dependent variable in our analysis representing the number of correctly solved items^2^.

The explanatory variables used in our analysis are grouped according to the overarching factors of the theoretical model: motivation, efficiency, and exposure.

As an indicator of motivation, we consider the parents' *intention to stay* in Germany. Using a dummy variable, we compare parents who want to stay in Germany forever (=1) with those who plan to leave Germany in the future (=0). Another condition associated with motivation concerns refugees' *residence status*. We distinguish between families with accepted residence permits (recognized as entitled to asylum or as refugees = 0) and parents with insecure residence statuses (e.g., toleration, decision on application still pending, obliged to leave the country), and thus more uncertain prospects of staying in Germany for a longer period (=1).

Children's efficiency of language learning is related to their *general cognitive abilities*. The measure of cognitive ability employed in the ReGES study is a matrices test (NEPS-MAT) with 12 items assessing figural reasoning (Lang et al., 2014; for details on its use in ReGES see FDZ-LIfBi, 2021). Test results are calculated in a sum score representing the number of tasks that each student solved correctly. Furthermore, we used the PROTECT scale (Boillat and Chamouton, 2013) for identifying children at *risk of post-traumatic stress disorder (PTSD)*. Parents were asked 10 questions about the mental and physical condition of the target child, for instance, whether the child often has nightmares or often has trouble concentrating. According to the scale, children who show less than four symptoms have low risk of PTSD, while children who are above this threshold have medium (4–7 symptoms) to high (8–10 symptoms) risk of PTSD. In the analysis, we differentiate between children at risk (medium/high = 1) and children who are not at risk (low = 0).

We examine several indicators for conditions that shape children's exposure to the German language. This includes the *duration of stay* (in months) as a broad measure of the overall language exposure. In addition, we operationalize *German language support in the family* based on specific learning strategies and materials that the parents use to promote their child's language acquisition, such as using language learning apps, watching children's TV programmes in German, or reading German children's books aloud. A sum score indicates the number of measures the parents regularly take to promote their child's learning, ranging from 0 to 6. The *German language competency level of parents* represent another condition for exposure within the family. Parents were asked how well they can understand, speak, read and write in German. The response scale ranged from not at all (=1) to very well (=5). We calculated the average of the four self-report questions and selected the score of the parent with the highest German proficiency. The parents were also asked how many hours the child spends on average on a normal weekday in situations in which they hear or speak German. This information serves as indicator of children's *German language contact* in everyday life. With regard to institutional language exposure, we consider whether the child currently receives *German language instruction* (no = 0/yes = 1) and *attends preschool* (no = 0/yes = 1). As a refugee-specific condition, we examine differences between children living in *collective accommodation* (=1), and children living in private accommodation (=0).

In addition to these explanatory variables, we control for the age (in months), gender, and country of origin of the child, as well as the highest educational level of the parents, distinguishing between primary education or below, secondary education, and tertiary education. At the regional level, the federal state, the population size of the municipality in which the children live, and the care rate^3^ of children aged three to six in the municipality are controlled for. Table 1 gives an overview of the distribution of all variables used in our analysis.

**Table 1:** Distribution of model variables.

**Variable**	**Range**	**Mean/%**	**SD**	**Missing (in %)**
German language competencies (PPVT-4)	[6–203]	56.54	29.56	0.00
**Motivation**				
Intention to stay long-term (in %)		84.70		2.98
Insecure residence status (in %)		16.34		11.93
**Efficiency**				
General cognitive abilities	[0–12]	6.83	2.87	5.29
Risk of PTSD (in %)		4.97		7.01
**Exposure**				
Duration of stay (in months)	[4–56]	28.43	8.77	0.00
German language support in family	[1–6]	2.10	1.22	1.19
Parents' German language competency level	[1–5]	3.50	0.75	0.07
German language contact (in hours/day)	[0–24]	5.53	3.38	2.01
German language instruction (in %)		27.65		2.39
Preschool attendance (in %)		84.86		0.97
Collective accommodation (in %)		10.29		0.00
**Controls**				
Age (in months)	[52–125]	68.69	11.13	0.00
Female (in %)		48.25		0.00
Country of origin (in %)				0.00
Syria		77.03		
Afghanistan		5.29		
Iraq		13.57		
Other		4.10		
Highest education of parents (in %)				0.60
None/primary		42.46		
Secondary		30.31		
Tertiary		27.23		
Federal state (in %)				0.00
Bavaria		8.72		
Hamburg		5.67		
North Rhine-Westphalia		68.83		
Rhineland-Palatinate		7.38		
Saxony		9.40		
Population size of municipality (in %)				0.00
5,000–99,999		7.23		
100,000–499,999		33.04		
500,000 an above		59.73		
Care rate in municipality		87.61	3.81	0.00

*N = 1,341 children; Source: doi: 10.5157/ReGES:RC1:SUF:2.0.0*.

### Methodological Approach

To analyze the relationship between German language competencies and the conditions discussed above, we estimate linear regression models. In a first step, we examine general, as well as refugee-specific conditions to identify the factors that significantly correlate with refugee children's German language competencies^4^. Since we are particularly interested in the interrelations between preschool attendance, institutional language instruction and German language acquisition, we further investigate whether the benefits of attending preschool are associated with the formal language instruction that the children receive within the institution, or with increased contact with the German language in everyday interactions. Therefore, we estimate stepwise regression models and examine how the correlation between preschool attendance and German language competencies changes if German language contact and/or language instruction are added to the model. In these models, a substantial decrease in the coefficient of preschool attendance could indicate a mediation through the respective condition that is controlled for. Moreover, we estimate the interaction term of preschool attendance and language instruction to disentangle influences of general preschool attendance from advantages that are specifically associated with language instruction within preschool. In a last step, we analyze whether the benefits of institutional support in preschool are particularly pronounced among children who have only limited exposure to the German language outside of the institutional context. We empirically address this question by estimating interaction terms between preschool attendance and three different indicators of exposure, namely German language support in the family, parents' German language competency levels, and children's daily contact with the German language.

We accounted for missing information concerning the independent variables and controls (see Table 1) with multiple imputation using iterated chained equations (White et al., 2011). Following the recommendations of von Hippel (2007, 2020), cases with missing values on the outcome variable were included in the imputation procedure and excluded from the analysis models. We applied a quadratic rule to determine the required number of imputations (*M* = 25), based on the fraction of missing information in our fully specified model (von Hippel, 2020). The descriptive results shown in Table 1 are based on the original data.

## Results

### The Conditions Associated With German Language Competencies of Refugee Children

In the first part of the analysis, we empirically address the assumption that refugee children respond to the same conditions that have been found to determine destination language acquisition in previous research. Table 2 shows the results of linear regressions of German language competencies on various general conditions (Model 1) and additional refugee-specific conditions (Model 2), all of which are linked to the three overarching factors of the general model of destination language acquisition (i.e., motivation, efficiency and exposure).

**Table 2:** Conditions of German language competencies among refugee children (linear regression).

	**Model 1**	**Model 2**
**Motivation**		
Intention to stay long-term	11.80^***^	11.84^***^
	(1.86)	(1.86)
Insecure residence status		−1.06
		(1.89)
**Efficiency**		
General cognitive abilities	2.68^***^	2.67^***^
	(0.27)	(0.27)
Risk of PTSD		0.70
		(3.86)
**Exposure**		
Duration of stay	0.60^***^	0.59^***^
	(0.09)	(0.09)
German language support in family	1.12^+^	1.16^*^
	(0.58)	(0.59)
Parents' German language competency level	5.40^***^	5.30^***^
	(1.10)	(1.10)
German language contact	0.52^*^	0.51^*^
	(0.26)	(0.26)
German language instruction	6.71^***^	6.70^***^
	(1.86)	(1.88)
Preschool attendance	4.45^*^	4.39^+^
	(2.25)	(2.24)
Collective accommodation		−1.44
		(2.87)
*R* ^2^	0.24	0.24

+ *p < 0.10*,

* *p < 0.05*,

** *p < 0.01*,

*** *p < 0.001; standard errors in parentheses; N = 1,341 children; additionally controlled for age, gender, country of origin, highest education of parents, federal state, population size of municipality, and care rate in municipality (for the full models including all coefficients see Supplementary Table 1). Source: doi: 10.5157/ReGES:RC1:SUF:2.0.0*.

The results show statistically significant associations between children's German language competencies and almost all indicators of motivation, efficiency and exposure that are included in Model 1. In line with the theoretical expectations, we find that children whose parents intend to stay long-term in Germany achieve higher German language competency levels than children from families that plan to leave Germany in the future, and thus, might have lower motivation to learn the destination language. With regard to learning efficiency, children's general cognitive abilities positively correlate with the German language competency levels they achieve. Moreover, the results concerning conditions that can be linked to exposure show a consistent pattern. German language competency levels of refugee children improve with the duration of stay in Germany. For example, children's competency levels increase on average by 7.2 points on the test score per year they stay in Germany (*b* = 0.60 per month). When it comes to German language support which children may receive in their families, we do not find a statistically significant association (*p* > 0.05), yet the coefficient points into the expected direction. Furthermore, higher German language competencies of parents, and regular German language contact in everyday life correlate positively with children's language competency levels. Among institutional factors, preschool attendance, and in particular, formal language instruction are significantly correlated with children's German language competency levels. For example, receiving German language instruction is associated with an average increase of 6.7 points on the test score, which approximates the average improvement in German language competencies that children achieve during a year in Germany, *ceteris paribus*.

Overall, the results corroborate the findings from prior research. It would therefore seem that the conditions that have been found to shape the language competency levels of other immigrant populations are also relevant among refugee children, and thus, appear to apply rather generally to the process of destination language acquisition. In Model 2, we examine whether additional conditions that are specifically associated with the situation of refugee children become relevant. We assumed that insecure residence status, and the risk of post-traumatic stress disorder represent unfavorable circumstances that hinder children's German language acquisition. Moreover, we hypothesized that living in collective accommodation has an impact upon the development of German language competencies, yet opposing theoretical arguments did not yield a clear prediction for the direction of this association. Contrary to the theoretical expectation, we do not find a statistically significant association of any of the three conditions with children's German language competency levels. Thus, refugee-specific aspects do not appear to make a substantial contribution to explaining German language acquisition among refugee children.

### Institutional Language Support in Preschool

Given the significance of formal language instruction found in our analysis, we further investigate whether attending preschool supports refugee children by proving language instruction for those who need it. Table 3 shows the results of the interrelation between preschool attendance, language instruction and German language competencies. In addition to the variables outlined in Table 3, the regression models include the same set of independent variables and controls used in the previous analysis (Table 2, Model 2). To differentiate possible influences of language instruction in preschool from other aspects associated with preschool attendance, we first analyze how the correlation between general preschool attendance and German language competencies changes if German language contact and/or German language instruction are additionally controlled for.

**Table 3:** Interrelation between preschool, language instruction, and German language competencies (linear regression).

	**Model 3**	**Model 4**	**Model 5**	**Model 6**	**Model 7**	**Model 8**	**Model 9**
Preschool attendance	6.91^**^	6.03^**^	5.41^*^	4.39^+^	3.40	3.40	
	(2.17)	(2.24)	(2.20)	(2.24)	(2.30)	(2.30)	
German language contact		0.47^+^		0.51^*^	0.52^*^	0.52^*^	0.29
		(0.26)		(0.26)	(0.26)	(0.26)	(0.29)
German language instruction			6.50^***^	6.70^***^	−9.34	−9.34	7.08^***^
			(1.89)	(1.88)	(8.90)	(8.90)	(1.91)
Preschool attendance * German					16.57^+^		
lang. instruction (*interaction*)					(9.03)		
Preschool attendance + German						19.97^*^	
lang. instruction (*combined*)						(8.74)	
*R* ^2^	0.23	0.24	0.24	0.24	0.25	0.25	0.21

+ *p < 0.10*,

* *p < 0.05*,

** *p < 0.01*,

*** *p <0.001; standard errors in parentheses; N = 1,341 children (Model 9: N = 1,127); additionally controlled for intention to stay, insecure residence status, general cognitive abilities, risk of PTSD, duration of stay, German language support in family, parents' German language competency level, collective accommodation, age, gender, country of origin, highest education of parents, federal state, population size of municipality, and care rate in municipality (for the full models including all coefficients see Supplementary Table 1). Source: doi: 10.5157/ReGES:RC1:SUF:2.0.0*.

Model 3 shows the ‘total’ correlation between preschool attendance and German language competencies. Refugee children who attend preschool perform significantly better in the language test (*b* = 6.91, *p* < 0.01) than their peers who do not go to preschool. This correlation is considerably reduced if we add children's German language contact (Model 4: *b* = 6.03, *p* < 0.01), and even more if we instead control for language instruction (Model 5: *b* = 5.41, *p* < 0.05). These relationships indicate that children in preschool benefit from both increased contact to the German language and the language instruction they receive within the preschool context; language instruction appears to be slightly more important in this regard. Both conditions independently support refugee children in their German language acquisition and account for a considerable part of the total correlation between preschool and language competencies (see Model 6)^5^.

Based on our data, it is difficult to disentangle the influences associated with general preschool attendance from those of additional language instruction, because the majority of children (97%) who receive language instruction also attend preschool. This finding suggests that formal language instruction of refugee children in Germany mainly takes place in preschool. However, while 85 percent of the children in our analysis sample attend preschool, less than a third (28%) actually receive language instruction (see Table 1). To further determine the relevance of language instruction in preschool for refugee children's German language acquisition, we estimate the interaction term of preschool attendance and language instruction (Model 7). In this model, the correlation between preschool attendance and German language competencies is estimated conditional on whether the children also receive language instruction or not. The so-called conditional main effect of preschool attendance represents the correlation between preschool attendance and German language competencies for children without language instruction, and the corresponding interaction term indicates the degree to which the correlation differs for children who also receive language instruction. The results reveal a clear pattern: preschool attendance without language instruction does not yield a significant advantage for language learning (*b* = 3.40, *p* > 0.10). Children who also receive language instruction, in contrast, appear to benefit greatly from attending preschool. Although the coefficient of the interaction term is substantially large in magnitude, it does not reach statistical significance at the 5 percent level (*b* = 16.57, *p* < 0.10). However, further analysis estimating the combined coefficient of preschool attendance and additional language instruction yields a statistically significant result (*b* = 3.40 + 16.57 = 19.97, *p* < 0.05; see Model 8)^6^. The average language improvement that children achieve as a result of language instruction in preschool is about five times greater compared to the improvement seen among children who attend preschool but do not receive language instruction (16.57/3.40 = 4.87; see Model 7). The results of the comparison between preschool children with and without language instruction indicate that formal language instruction is the main driver in promoting German language competencies among refugee children in the context of preschool. In order to more accurately assess the magnitude of potential benefits associated with language instruction *in preschool*, we estimated the baseline model (Model 2 and 6, respectively) in a separate analysis only for children who attend preschool (Model 9). The net coefficient of language instruction in preschool (*b* = 7.08, *p* < 0.001) does not substantially differ from the results for the total population; probably because in the analysis sample, only very few children who do not attend preschool receive language instruction (*N* = 9).

Finally, we investigate whether the advantages associated with preschool attendance differ with regard to children's language exposure outside of the institutional context. We assumed that refugee children who have only limited exposure to the German language at home and in their everyday lives should particularly benefit from attending preschool. Table 4 presents the results of the correlation between preschool attendance and German language competencies conditional on the German language support that children receive in their family, the parents' level of German language competencies, and children's daily German language contact. In line with the theoretical expectations, we find a negative and significant interaction term of preschool attendance in combination with German language support in the family (Model 10), as well as with German language contact (Model 12). In these models, the conditional main effects of preschool attendance (*b* = 13.13, *p* < 0.001 and *b* = 11.30, *p* < 0.001) are considerably higher compared to the unconditional coefficient (*b* = 4.39, *p* < 0.10; see Model 2 in Table 2). Accordingly, attending preschool has the biggest impact for children who do not receive language support at home, and who generally have limited contact to the German language in their everyday lives. The negative interaction terms indicate that with increasing language support and contact, the benefits associated with preschool attendance decrease. Thus, institutional language support in preschool can compensate for lacking German language exposure at home and in everyday life. With regard to the German language competency levels of parents, we do not find such a pattern; i.e., children benefit from preschool attendance irrespective of whether their parents speak German (well) or not (see Model 11).

**Table 4:** Correlation between preschool attendance and German language competencies conditional on children's German language exposure at home and in everyday live (linear regression).

	**Model 10**	**Model 11**	**Model 12**
German language support in family	5.10^***^	1.17^*^	1.15^*^
	(1.30)	(0.59)	(0.59)
Parents' German language competency level	5.29^***^	5.94^*^	5.50^***^
	(1.09)	(2.63)	(1.10)
German language contact	0.52^*^	0.52^*^	1.92^***^
	(0.26)	(0.26)	(0.52)
Preschool attendance	13.13^***^	7.01	11.30^***^
	(3.72)	(9.31)	(3.23)
**Interactions**			
Preschool attendance* German language	−4.66^***^		
support in family	(1.41)		
Preschool attendance* Parents' German		−0.79	
language competency level		(2.72)	
Preschool attendance * German language			−1.66^**^
contact			(0.58)
*R* ^2^	0.25	0.24	0.25

+ *p < 0.10*,

* *p < 0.05*,

** *p < 0.01*,

*** *p < 0.001; standard errors in parentheses; N = 1,341 children; additionally controlled for intention to stay, insecure residence status, general cognitive abilities, risk of PTSD, duration of stay, German language instruction, collective accommodation, age, gender, country of origin, highest education of parents, federal state, population size of municipality, and care rate in municipality (for the full models including all coefficients see Supplementary Table 1). Source: doi: 10.5157/ReGES:RC1:SUF:2.0.0*.

## Discussion

In this article, we investigated the conditions that foster or hinder the acquisition of destination language competencies among young refugee children in Germany. Apart from identifying relevant conditions in general, we were specifically interested in the role that preschool attendance and formal language instruction play in children's German language acquisition. Since refugee children typically learn the destination language from scratch upon arrival in the destination country, and often have limited exposure to that language in their everyday lives, we expected that they would benefit greatly from institutional language support.

Analyzing data from the ReGES study, we find that refugee children respond to the same conditions that were found in prior research to determine destination language competencies of immigrant children. This finding is in line with the results of previous studies comparing the conditions of German language acquisition between adult refugees and other new immigrants (Kosyakova et al., 2021; Kristen and Seuring, 2021). Thus, the process of destination language acquisition appears to follow regularities that apply to all immigrants (including refugees) in the same way. Consistent with this view, the results of our study indicate empirical support for the general theoretical model of destination language acquisition (Chiswick and Miller, 1995, 2001; Esser, 2006a,b) and its application to refugee children. We find that conditions associated with all three theoretical factors—motivation, efficiency, and exposure—are relevant for the destination language learning of refugee children, with exposure—particularly in the institutional context of preschool education—being key for this specific population.

Moreover, we investigated additional conditions associated with the specific circumstances that refugees often experience, including possible consequences of insecure residence status, risk of post-traumatic stress disorders, and living in collective accommodation. None of these refugee-specific conditions were significantly associated with German language competencies in our analysis, suggesting that the ‘standard’ set of conditions typically addressed in the general theoretical model of destination language acquisition also hold for refugee children without the need to extend the model. Whereas for the experience of traumatic events and related health issues, previous studies often also have not found a (negative) association with destination language competencies (e.g., Hunkler and Khourshed, 2020; Bernhard and Bernhard, 2021; Kosyakova et al., 2021; Kristen and Seuring, 2021), insecure residence status (e.g., Kristen and Seuring, 2021), and living in collective accommodation (e.g., Kosyakova et al., 2021) have been shown to hinder destination language acquisition among adult refugees. Our results indicate that these legal and living conditions might be less relevant among refugee children, possibly because children have contact with the destination language in their everyday lives, for instance, in (pre)school, irrespective of these circumstances. In addition, children may be less aware of the uncertainty of their chances of staying in the host country. Nevertheless, revealing that the unfavorable conditions typically associated with refugees' specific situation do not significantly hinder refugee children in acquiring the destination language represents a key finding of our contribution. Whereas we expect this finding to apply to the majority of refugee children living in Germany, it is important to note that the legal and living conditions might still be relevant under certain circumstances, for instance, among refugees whose asylum application has been rejected and who must live in special accommodation until leaving the country.

Our findings emphasize the importance of preschool attendance and formal language instruction. In line with prior studies consistently showing that immigrant children benefit particularly from attending preschool (e.g., Sammons et al., 2004; Magnuson et al., 2006; Loeb et al., 2007; Gormley, 2008; Burger, 2012; Becker et al., 2013; Klein and Becker, 2017), we find a similar pattern for refugee children. In addition to the exposure to the destination language that children experience in daily interactions during general care, preschools provide access to formal language instruction, which we identify as one of the most important factors in promoting refugee children's German language acquisition. Furthermore, the results show that the advantages associated with language support in preschool are particularly pronounced among refugee children who have only limited exposure to the German language at home and in their everyday lives. Attending preschool could thus be an effective route in supporting the destination language acquisition of refugee children, and especially of those in need. However, 85 percent of the refugee children in our sample attended preschool, whereas only about 28% of the children received formal language instruction. This discrepancy points to a possible imbalance between the demand for and provision of institutional language support in preschool. It is unclear, however, whether too few opportunities for language instruction are provided in institutions, or whether refugee children do not take full advantage of the support offered in preschool, for example, because their parents may not recognize their children's need for this support, or are not aware of the services on offer. Prior findings indicating that most refugee parents assess their children's German language competencies to be good or very good (see Will et al., 2018) could lend support to the explanation that at least some parents do not see the necessity of their children receiving language instruction.

In our study, we examined the correlation between German language competencies and various conditions that are assumed to affect language acquisition. As we employ a cross-sectional analysis, we cannot make causal claims regarding the processes underlying destination language acquisition. The design also does not allow us to account for potential selection bias that could affect the results. For example, usually only children with language difficulties receive additional language instruction in preschool (e.g., Becker et al., 2016). Accordingly, if it is primarily the children with lower German language competency levels in the sample who receive language instruction, we will have underestimated the ‘effects’ of preschool attendance and language instruction. However, it can be expected that the majority of refugee children had rather low German language competency levels at the time of the survey, because they had only recently immigrated to Germany and started to learn German. Selective participation in the language test could be another source for bias. Refugee children who could not follow the test instructions in one of the administration languages (Arabic, German, English, and Kurmanji), as well as those who did not pass the first set of tasks in the test, did not achieve a valid test score and were consequently excluded from the analysis. Considering that we excluded children who hardly speak any German at all, and who presumably would particularly benefit from language support in preschool, it may well be the case that the advantages associated with preschool attendance and language instruction might be even more pronounced than indicated by our analysis.

In our analysis, we focused on whether refugee children attend preschool or take language instruction in general. We were able to show how important attending preschool is for the acquisition of German language competencies, and the crucial role that language instruction plays in this process. Building on these findings, however, the role of preschools and language instruction could be explored in more detail in future. Further aspect of these measures could be also relevant, for example, the duration of preschool attendance or the timing, duration, and extent of language instruction. Furthermore, the finding that language support in preschool is particularly beneficial for refugee children who lack exposure to the destination language outside the institutional context, raises the question of whether the children who are most in need actually are the ones who receive language instruction. These questions should be addressed in future research to complement the findings of our study.

Overall, our results emphasize the importance of language instruction in preschool for the destination language acquisition of refugee children. In light of findings indicating that formal instruction is most efficient in an early stage of the learning process (e.g., Hoehne and Michalowski, 2016; Bernhard and Bernhard, 2021), facilitating early access to preschool for refugee children could constitute a measure to support their destination language acquisition in the long term. This becomes even more important since refugee parents often report that the main reason for their children not attending preschool is that they could not find them a place (see Will et al., 2018). At the same time, however, it must be ensured that children who need language support actually also receive this support. This means two different target points could be addressed. On the one hand, the range of language instruction within the institutions would have to be significantly expanded. On the other hand, it could be useful to employ additional strategies to inform refugee parents about the opportunities and benefits of language instruction for their children.

## Data Availability Statement

Publicly available datasets were analyzed in this study. This data can be found here: https://doi.org/10.5157/ReGES:RC1:SUF:2.0.0. The Stata code used to operationalize and analyze the data can be found in the Supplementary Material (online) and may be used without restriction (for replication).

## Author Contributions

All authors listed have made a substantial, direct, and intellectual contribution to the work and approved it for publication.

## Funding

The data we use for our research were collected in the framework of the project ReGES—Refugees in the German educational system. This project was funded by the German Federal Ministry of Education and Research (BMBF) under grant number FLUCHT03. The present work took place partly within the framework of the project Educational Trajectories of Refugee Children and Adolescents, which was funded by the BMBF under grant number FLUCHT2021. The open access publication of this article was funded by the Open Access Fund of the Leibniz Association. The content of this publication is solely the responsibility of the authors.

## Conflict of Interest

The authors declare that the research was conducted in the absence of any commercial or financial relationships that could be construed as a potential conflict of interest.

## Publisher's Note

All claims expressed in this article are solely those of the authors and do not necessarily represent those of their affiliated organizations, or those of the publisher, the editors and the reviewers. Any product that may be evaluated in this article, or claim that may be made by its manufacturer, is not guaranteed or endorsed by the publisher.

## Footnotes

^1^This paper uses data from the project “Refugees in the German Educational System” (ReGES): Refugee Cohort 1—Children, doi: 10.5157/ReGES:RC1:SUF:2.0.0, which was funded by the German Ministry of Education and Research (BMBF) under grant number FLUCHT03 and conducted by the Leibniz Institute for Educational Trajectories (LIfBi).

^2^In the ReGES study, a German grammar test was employed in addition to the vocabulary test, namely the “Test of Reception of Grammar” (TROG; Bishop, 1989) in its German version (TROG-D; Fox-Boyer, 2016; for details on its use in ReGES see FDZ-LIfBi, 2021). To check the robustness of our findings, we replicated the analyses using the scores of the grammar test. The analysis of children's German grammar competencies yielded similar results (see Supplementary Table 3).

^3^The care rate indicates the share of children who attend childcare of all children in the respective age group. We gathered the data on regional care rates for the year 2017 from the Federal Statistical Office of Germany.

^4^In light of the large number of independent variables—associated with the three theoretical constructs (motivation, efficiency, and exposure)—included in the model, some of which may be highly correlated with each other, we estimated the variance inflation factors (VIF) to check for multicollinearity amongst the independent variables. All independent variables showed a high degree of tolerance (0.87–0.98), indicating that multicollinearity does not (substantially) bias the results (see Supplementary Table 2).

^5^Model 6 is equivalent to Model 2 in Table 2. The results were also included in Table 3 as a reference to allow the coefficient of preschool attendance to be compared across different model specifications.

^6^The difference between these two models lies in the reference category used in the analysis: the interaction coefficient in Model 7 indicates that the average German language competency level of children who both attend preschool and receive language instruction differs from those of children who attend preschool without language instruction. The combined coefficient in Model 8, in contrast, compares children attending preschool and receiving language instruction with those children who neither attend preschool nor receive language instruction.
