# Supplementary material for: German Language Acquisition of Refugee Children—The Role of Preschools and Language Instruction
Source: Front Sociol. 2022 May 9;7:840696. doi: 10.3389/fsoc.2022.840696 (PMC9125026; doi:10.3389/fsoc.2022.840696)
Supplement: Supplementary file 1 [file Table_1.pdf]

Supplementary Material

Supplementary Table 1. Coefficients of all variables included in regression models of Tables 2, 3 and 4

	Model 1	Model 2	Model 3	Model 4	Model 5	Model 6	Model 7	Model 8	Model 9	Model 10	Model 11	Model 12
Motivation												
Intention to stay long-term	11.80***	11.84***	11.52***	11.51***	11.84***	11.84***	11.70***	11.70***	11.85***	12.01***	11.84***	11.87***
	(1.86)	(1.86)	(1.87)	(1.84)	(1.89)	(1.86)	(1.87)	(1.87)	(2.03)	(1.86)	(1.86)	(1.87)
Insecure residence status		-1.06	-1.15	-1.11	-1.11	-1.06	-1.00	-1.00	-0.88	-0.94	-1.10	-0.98
		(1.89)	(1.90)	(1.89)	(1.90)	(1.89)	(1.89)	(1.89)	(2.06)	(1.89)	(1.89)	(1.90)
Efficiency												
General cognitive abilities	2.68***	2.67***	2.68***	2.68***	2.68***	2.67***	2.67***	2.67***	2.48***	2.62***	2.67***	2.63***
	(0.27)	(0.27)	(0.27)	(0.27)	(0.27)	(0.27)	(0.27)	(0.27)	(0.30)	(0.27)	(0.27)	(0.27)
Risk of PTSD		0.70	1.60	1.74	0.57	0.70	0.88	0.88	0.51	0.37	0.67	0.66
		(3.86)	(3.79)	(3.77)	(3.88)	(3.86)	(3.87)	(3.87)	(4.08)	(3.87)	(3.86)	(3.86)
Exposure												
Duration of stay	0.60***	0.59***	0.60***	0.58***	0.61***	0.59***	0.59***	0.59***	0.55***	0.59***	0.59***	0.58***
	(0.09)	(0.09)	(0.09)	(0.09)	(0.09)	(0.09)	(0.09)	(0.09)	(0.09)	(0.09)	(0.09)	(0.09)
German language support in family	1.12+	1.16*	1.08+	0.97+	1.28*	1.16*	1.16*	1.16*	0.53	5.10***	1.17*	1.15*
	(0.58)	(0.59)	(0.58)	(0.58)	(0.58)	(0.59)	(0.59)	(0.59)	(0.64)	(1.30)	(0.59)	(0.59)
Parents' German language competency level	5.40***	5.30***	6.03***	5.93***	5.43***	5.30***	5.34***	5.34***	5.21***	5.29***	5.94*	5.50***
	(1.10)	(1.10)	(1.10)	(1.10)	(1.10)	(1.10)	(1.10)	(1.10)	(1.15)	(1.09)	(2.63)	(1.10)
German language contact	0.52*	0.51*		0.47+		0.51*	0.52*	0.52*	0.29	0.52*	0.52*	1.92***
	(0.26)	(0.26)		(0.26)		(0.26)	(0.26)	(0.26)	(0.29)	(0.26)	(0.26)	(0.52)
German language instruction	6.71***	6.70***			6.50***	6.70***	-9.34	-9.34	7.08***	6.68***	6.73***	6.52***
	(1.86)	(1.88)			(1.89)	(1.88)	(8.90)	(8.90)	(1.91)	(1.88)	(1.88)	(1.87)

Supplementary Table 1 continued.

	Model 1	Model 2	Model 3	Model 4	Model 5	Model 6	Model 7	Model 8	Model 9	Model 10	Model 11	Model 12
Preschool attendance	4.45*	4.39+	6.91**	6.03**	5.41*	4.39+	3.40	3.40		13.13***	7.01	11.30***
	(2.25)	(2.24)	(2.17)	(2.24)	(2.20)	(2.24)	(2.30)	(2.30)		(3.72)	(9.31)	(3.23)
Collective accommodation		-1.44	-1.50	-1.21	-1.76	-1.44	-1.51	-1.51	-4.13	-1.90	-1.42	-1.83
		(2.87)	(2.94)	(2.85)	(2.96)	(2.87)	(2.87)	(2.87)	(3.16)	(2.85)	(2.86)	(2.84)
Interactions												
Preschool attendance * German language instruction							16.57+					
							(9.03)					
Preschool attendance + German language instruction (*combined coefficient*)								19.97*				
								(8.74)				
Preschool attendance * German language support in family										-4.66***		
										(1.41)		
Preschool attendance * Parents' German language competency level											-0.79	
											(2.72)	
Preschool attendance * German language contact												-1.66**
												(0.58)
Controls												
Age	0.32***	0.32***	0.34***	0.32***	0.34***	0.32***	0.31***	0.31***	0.30**	0.27***	0.32***	0.28***
	(0.08)	(0.08)	(0.08)	(0.08)	(0.08)	(0.08)	(0.08)	(0.08)	(0.10)	(0.08)	(0.08)	(0.08)
Female	1.34	1.34	1.52	1.55	1.31	1.34	1.38	1.38	1.37	1.44	1.33	1.51
	(1.46)	(1.47)	(1.47)	(1.47)	(1.47)	(1.47)	(1.46)	(1.46)	(1.59)	(1.46)	(1.47)	(1.46)
Country of origin (ref. *Syria*)												
Afghanistan	1.55	1.86	3.20	2.75	2.37	1.86	2.40	2.40	3.37	2.01	1.82	2.45
	(3.01)	(3.08)	(3.12)	(3.10)	(3.11)	(3.08)	(3.11)	(3.11)	(3.36)	(3.07)	(3.07)	(3.12)
Iraq	-0.98	-0.88	-0.85	-1.15	-0.56	-0.88	-0.72	-0.72	-2.10	-0.65	-0.88	-0.85
	(2.26)	(2.28)	(2.27)	(2.27)	(2.28)	(2.28)	(2.27)	(2.27)	(2.42)	(2.26)	(2.28)	(2.26)
Other	-0.06	0.33	0.99	0.56	0.80	0.33	0.56	0.56	1.42	0.34	0.32	0.41
	(3.64)	(3.75)	(3.71)	(3.66)	(3.80)	(3.75)	(3.78)	(3.78)	(4.25)	(3.75)	(3.76)	(3.79)

Supplementary Table 1 continued.

	Model 1	Model 2	Model 3	Model 4	Model 5	Model 6	Model 7	Model 8	Model 9	Model 10	Model 11	Model 12
Highest education of parents (*ref. None/primary*)												
Secondary	-3.88*	-3.85*	-3.52+	-3.59+	-3.77*	-3.85*	-3.95*	-3.95*	-4.55*	-3.91*	-3.83*	-3.75*
	(1.84)	(1.84)	(1.86)	(1.85)	(1.85)	(1.84)	(1.84)	(1.84)	(2.00)	(1.84)	(1.85)	(1.84)
Tertiary	-2.42	-2.47	-2.04	-1.93	-2.58	-2.47	-2.53	-2.53	-2.26	-2.48	-2.43	-2.37
	(2.09)	(2.10)	(2.11)	(2.12)	(2.10)	(2.10)	(2.10)	(2.10)	(2.25)	(2.09)	(2.10)	(2.09)
Federal state (*ref. North Rhine-Westphalia*)												
Bavaria	3.98	4.11	4.98+	4.51	4.64+	4.11	4.06	4.06	2.41	3.50	4.04	3.84
	(2.81)	(2.82)	(2.82)	(2.84)	(2.80)	(2.82)	(2.82)	(2.82)	(3.10)	(2.79)	(2.82)	(2.79)
Hamburg	-3.53	-2.58	0.15	-1.16	-1.11	-2.58	-2.55	-2.55	-3.09	-2.75	-2.60	-2.28
	(3.40)	(3.97)	(4.07)	(3.81)	(4.28)	(3.97)	(3.95)	(3.95)	(4.39)	(3.95)	(3.96)	(4.00)
Rhineland-Palatinate	9.48**	9.57**	9.22**	9.57**	9.18*	9.57**	9.72**	9.72**	8.12*	9.46**	9.56**	9.39**
	(3.62)	(3.62)	(3.58)	(3.60)	(3.59)	(3.62)	(3.63)	(3.63)	(3.82)	(3.64)	(3.63)	(3.61)
Saxony	-7.70***	-7.75***	-8.71***	-8.73***	-7.76***	-7.75***	-7.76***	-7.76***	-8.89***	-7.77***	-7.75***	-7.41**
	(2.32)	(2.33)	(2.30)	(2.30)	(2.34)	(2.33)	(2.34)	(2.34)	(2.59)	(2.32)	(2.33)	(2.34)
Population size of municipality (*ref. 5,000 to 99,999*)												
100,000 to 499,999	-3.67	-3.98	-3.70	-3.22	-4.49	-3.98	-3.95	-3.95	-3.22	-3.64	-3.97	-3.58
	(3.21)	(3.29)	(3.36)	(3.37)	(3.28)	(3.29)	(3.29)	(3.29)	(3.32)	(3.32)	(3.30)	(3.28)
500,000 an above	-2.80	-2.93	-2.83	-2.69	-3.07	-2.93	-2.98	-2.98	-2.02	-2.98	-2.92	-2.68
	(3.24)	(3.34)	(3.39)	(3.40)	(3.32)	(3.34)	(3.33)	(3.33)	(3.34)	(3.36)	(3.34)	(3.32)
Care rate in municipality	0.62**	0.63**	0.75**	0.70**	0.69**	0.63**	0.62**	0.62**	0.76**	0.65**	0.63**	0.60*
	(0.24)	(0.24)	(0.24)	(0.24)	(0.24)	(0.24)	(0.24)	(0.24)	(0.26)	(0.24)	(0.24)	(0.24)
Constant	-90.47***	-90.35***	-103.52***	-98.12***	-96.47***	-90.35***	-88.79***	-88.79***	-90.92***	-96.71***	-93.00***	-91.41***
	(21.97)	(22.10)	(22.03)	(22.08)	(22.10)	(22.10)	(22.20)	(22.20)	(24.54)	(22.13)	(23.90)	(22.02)
R^2	0.24	0.24	0.23	0.24	0.24	0.24	0.25	0.25	0.21	0.25	0.24	0.25

*+ p<0.10, * p<0.05, ** p<0.01, *** p<0.001; standard errors in parentheses; N=1,341 children (Model 9: N=1,127); Source: doi:10.5157/ReGES:RC1:SUF:2.0.0.*

Supplementary Table 2. Variance inflation factors (VIF) of independent variables (model specification according to Model 2, Table 2)

	VIF	Tolerance (1/VIF)
Preschool attendance	1.15	0.87
Collective accommodation	1.13	0.88
German language instruction	1.11	0.90
General cognitive abilities	1.10	0.91
German language contact	1.09	0.92
Duration of stay	1.09	0.92
Parents' German language competency level	1.09	0.92
German language support in family	1.07	0.93
Intention to stay long-term	1.03	0.97
Insecure residence status	1.03	0.97
Risk of PTSD	1.02	0.98

N=1,341 children; Source: doi:10.5157/ReGES:RC1:SUF:2.0.0.

Supplementary Table 3. Results of robustness analysis using children's German grammar competencies (scores from TROG-D) instead of vocabulary competencies (scores from PPVT-4) as dependent variable

	Model 1	Model 2	Model 3	Model 4	Model 5	Model 6	Model 7	Model 8	Model 9	Model 10	Model 11	Model 12
Motivation												
Intention to stay long-term	4.18***	4.13***	4.07***	4.05***	4.14***	4.13***	4.12***	4.12***	4.39***	4.22***	4.13***	4.12***
	(0.85)	(0.85)	(0.85)	(0.85)	(0.85)	(0.85)	(0.85)	(0.85)	(0.96)	(0.84)	(0.85)	(0.85)
Insecure residence status		0.73	0.83	0.82	0.75	0.73	0.80	0.80	0.50	0.76	0.71	0.76
		(1.03)	(1.03)	(1.02)	(1.03)	(1.03)	(1.02)	(1.02)	(1.10)	(1.03)	(1.03)	(1.02)
Efficiency												
General cognitive abilities	1.60***	1.60***	1.61***	1.61***	1.60***	1.60***	1.60***	1.60***	1.53***	1.54***	1.60***	1.58***
	(0.13)	(0.13)	(0.13)	(0.13)	(0.13)	(0.13)	(0.13)	(0.13)	(0.14)	(0.13)	(0.13)	(0.13)
Risk of PTSD		-1.09	-0.70	-0.67	-1.12	-1.09	-1.05	-1.05	-1.07	-1.32	-1.10	-1.09
		(1.55)	(1.53)	(1.53)	(1.55)	(1.55)	(1.55)	(1.55)	(1.62)	(1.55)	(1.55)	(1.52)
Exposure												
Duration of stay	0.25***	0.25***	0.25***	0.24***	0.26***	0.25***	0.25***	0.25***	0.26***	0.26***	0.25***	0.24***
	(0.04)	(0.04)	(0.04)	(0.04)	(0.04)	(0.04)	(0.04)	(0.04)	(0.05)	(0.04)	(0.04)	(0.04)
German language support in family	1.30***	1.29***	1.33***	1.28***	1.35***	1.29***	1.29***	1.29***	0.71*	4.39***	1.30***	1.29***
	(0.30)	(0.30)	(0.30)	(0.30)	(0.30)	(0.30)	(0.30)	(0.30)	(0.32)	(0.67)	(0.30)	(0.30)
Parents' German language competency level	1.99***	2.01***	2.23***	2.19***	2.06***	2.01***	2.05***	2.05***	1.91**	1.99***	2.30*	2.14***
	(0.51)	(0.51)	(0.51)	(0.51)	(0.51)	(0.51)	(0.51)	(0.51)	(0.57)	(0.50)	(1.12)	(0.51)
German language contact	0.24*	0.24*		0.23*		0.24*	0.25*	0.25*	0.07	0.23*	0.25*	1.16***
	(0.11)	(0.11)		(0.11)		(0.11)	(0.11)	(0.11)	(0.12)	(0.11)	(0.11)	(0.24)
German language instruction	2.71**	2.73**			2.69**	2.73**	-1.53	-1.53	3.07***	2.80***	2.74**	2.67**
	(0.85)	(0.85)			(0.85)	(0.85)	(3.11)	(3.11)	(0.87)	(0.85)	(0.85)	(0.85)

Supplementary Table 3 continued.

	Model 1	Model 2	Model 3	Model 4	Model 5	Model 6	Model 7	Model 8	Model 9	Model 10	Model 11	Model 12
Preschool attendance	5.00***	4.98***	6.02***	5.58***	5.44***	4.98***	4.56***	4.56***		11.68***	6.18	9.50***
	(0.99)	(1.00)	(0.95)	(0.99)	(0.97)	(1.00)	(1.04)	(1.04)		(1.57)	(4.06)	(1.41)
Collective accommodation		0.03	-0.04	0.06	-0.07	0.03	0.03	0.03	-1.28	-0.54	0.03	-0.23
		(1.35)	(1.37)	(1.35)	(1.37)	(1.35)	(1.35)	(1.35)	(1.60)	(1.36)	(1.35)	(1.32)
Interactions												
Preschool attendance * German language instruction							4.53					
							(3.18)					
Preschool attendance + German language instruction (*combined coefficient*)								9.09**				
								(3.06)				
Preschool attendance * German language support in family										-3.73***		
										(0.72)		
Preschool attendance * Parents' German language competency level											-0.36	
											(1.23)	
Preschool attendance * German language contact												-1.11***
												(0.26)
Controls												
Age	0.23***	0.23***	0.24***	0.23***	0.24***	0.23***	0.23***	0.23***	0.21***	0.21***	0.23***	0.21***
	(0.03)	(0.03)	(0.04)	(0.04)	(0.03)	(0.03)	(0.03)	(0.03)	(0.04)	(0.03)	(0.03)	(0.04)
Female	1.76**	1.76**	1.77**	1.76**	1.77**	1.76**	1.76**	1.76**	2.00**	1.80**	1.75**	1.86**
	(0.66)	(0.66)	(0.67)	(0.67)	(0.66)	(0.66)	(0.66)	(0.66)	(0.75)	(0.66)	(0.66)	(0.66)
Country of origin (ref. *Syria*)												
Afghanistan	-0.73	-0.72	-0.15	-0.38	-0.48	-0.72	-0.54	-0.54	-0.45	-0.44	-0.74	-0.54
	(1.44)	(1.44)	(1.43)	(1.44)	(1.43)	(1.44)	(1.45)	(1.45)	(1.69)	(1.43)	(1.44)	(1.48)
Iraq	0.52	0.49	0.58	0.39	0.69	0.49	0.55	0.55	-0.03	0.60	0.49	0.52
	(1.05)	(1.06)	(1.05)	(1.05)	(1.06)	(1.06)	(1.06)	(1.06)	(1.18)	(1.04)	(1.06)	(1.04)
Other	-2.17	-2.32	-2.09	-2.23	-2.18	-2.32	-2.32	-2.32	-2.00	-2.21	-2.33	-2.19
	(1.56)	(1.61)	(1.62)	(1.59)	(1.64)	(1.61)	(1.61)	(1.61)	(1.85)	(1.63)	(1.61)	(1.63)

Supplementary Table 3 continued.

	Model 1	Model 2	Model 3	Model 4	Model 5	Model 6	Model 7	Model 8	Model 9	Model 10	Model 11	Model 12
Highest education of parents (*ref. None/primary*)												
Secondary	-0.59	-0.60	-0.44	-0.49	-0.55	-0.60	-0.63	-0.63	-0.91	-0.66	-0.60	-0.57
	(0.83)	(0.83)	(0.84)	(0.83)	(0.84)	(0.83)	(0.83)	(0.83)	(0.94)	(0.82)	(0.83)	(0.82)
Tertiary	0.63	0.65	0.84	0.85	0.64	0.65	0.62	0.62	1.06	0.59	0.66	0.65
	(1.00)	(1.00)	(1.01)	(1.01)	(1.00)	(1.00)	(1.00)	(1.00)	(1.09)	(0.99)	(1.00)	(0.99)
Federal state (*ref. North Rhine-Westphalia*)												
Bavaria	3.27*	3.32*	3.62**	3.35*	3.60**	3.32*	3.34*	3.34*	1.60	2.56*	3.29*	3.17*
	(1.30)	(1.32)	(1.32)	(1.31)	(1.33)	(1.32)	(1.31)	(1.31)	(1.46)	(1.30)	(1.31)	(1.29)
Hamburg	-5.39***	-5.34**	-4.12*	-4.53**	-4.91**	-5.34**	-5.25**	-5.25**	-5.44**	-5.15**	-5.35**	-5.20**
	(1.35)	(1.65)	(1.62)	(1.62)	(1.66)	(1.65)	(1.66)	(1.66)	(1.85)	(1.65)	(1.65)	(1.64)
Rhineland-Palatinate	4.78**	4.79**	4.47**	4.70**	4.55**	4.79**	4.84**	4.84**	4.27*	4.55**	4.77**	4.74**
	(1.70)	(1.71)	(1.70)	(1.71)	(1.69)	(1.71)	(1.71)	(1.71)	(1.82)	(1.72)	(1.71)	(1.70)
Saxony	-3.10**	-3.05*	-3.46**	-3.40**	-3.12**	-3.05*	-2.96*	-2.96*	-3.13*	-3.20**	-3.05*	-2.54*
	(1.19)	(1.19)	(1.20)	(1.20)	(1.20)	(1.19)	(1.19)	(1.19)	(1.40)	(1.17)	(1.19)	(1.20)
Population size of municipality (*ref. 5,000 to 99,999*)												
100,000 to 499,999	-3.09*	-2.95+	-3.00+	-2.75+	-3.20*	-2.95+	-2.95+	-2.95+	-2.39	-2.66+	-2.95+	-2.77+
	(1.50)	(1.54)	(1.55)	(1.55)	(1.54)	(1.54)	(1.53)	(1.53)	(1.61)	(1.52)	(1.54)	(1.51)
500,000 an above	-2.54+	-2.45+	-2.54+	-2.46+	-2.52+	-2.45+	-2.48+	-2.48+	-1.66	-2.42+	-2.45+	-2.33
	(1.45)	(1.47)	(1.49)	(1.49)	(1.47)	(1.47)	(1.47)	(1.47)	(1.54)	(1.46)	(1.48)	(1.45)
Care rate in municipality	0.13	0.13	0.19+	0.16	0.16	0.13	0.13	0.13	0.24+	0.16	0.13	0.10
	(0.11)	(0.11)	(0.12)	(0.12)	(0.11)	(0.11)	(0.11)	(0.11)	(0.13)	(0.11)	(0.11)	(0.11)
Constant	-34.51***	-34.71***	-40.45***	-37.83***	-37.48***	-34.71***	-34.01**	-34.01**	-35.48**	-41.24***	-35.97**	-33.84**
	(10.40)	(10.47)	(10.58)	(10.60)	(10.47)	(10.47)	(10.47)	(10.47)	(11.94)	(10.22)	(11.30)	(10.47)
R^2	0.30	0.30	0.29	0.29	0.30	0.30	0.30	0.30	0.23	0.31	0.30	0.31

*+ p<0.10, * p<0.05, ** p<0.01, *** p<0.001; standard errors in parentheses; N=1,683 children (Model 9: N=1,344); Source: doi:10.5157/ReGES:RC1:SUF:2.0.0.*
